# Functional Evaluations in the Monitoring of the River Ecosystem Processes: The Adige River as a Case Stu

**DOI:** 10.1100/tsw.2002.114

**Published:** 2002-03-12

**Authors:** M.G. Braioni, G. Salmoiraghi, F. Bracco, M. Villani, A. Braioni, L. Girelli

**Affiliations:** Department of Biology, University of Padova, Via U. Bassi 58/B, Italy; Department of Evolutionary Biology, University of Bologna, Via Selmi 3, Italy; 3Architect, vc. Ponte Nuovo 9, 37126 Verona, Italy

**Keywords:** river basin planning, river ecological processes, assessment methods, banks ecological indices, environmental landscape index, wild state index, buffer strip index

## Abstract

A model of analysis and environmental evaluation was applied to 11 stretches of the Adige River, where an innovative procedure was carried out to interpret ecological results. Within each stretch, the most suitable methods were used to assess the quality and processes of flood plains, banks, water column, bed, and interstitial environment. Indices were applied to evaluate the wild state and ecological quality of the banks (wild state index, buffer strip index) and the landscape quality of wide areas of the fluvial corridor (environmental landscape index). The biotic components (i.e., macrozoobenthos, phytoplankton and zooplankton, interstitial hyporheic fauna, vegetation in the riparian areas) were analysed by both quantitative and functional methods (as productivity, litter – processing and colonisation). The results achieved were then translated into five classes of functional evaluation. These qualitative assessments have thus preserved a high level of precision and sensitivity in quantifying both the quality of the environmental conditions and the integrity of the ecosystem processes. Read together with urban planning data, they indicate what actions are needed to restore and rehabilitate the Adige River corridor.

### INTRODUCTION

Four factors — the river continuum concept[1,2], nutrient spiralling[3], river mosaic[4,5], and a multidimensional approach to the river ecosystem[6,7,8] — have favoured the development of methods appropriate for measuring functional processes.

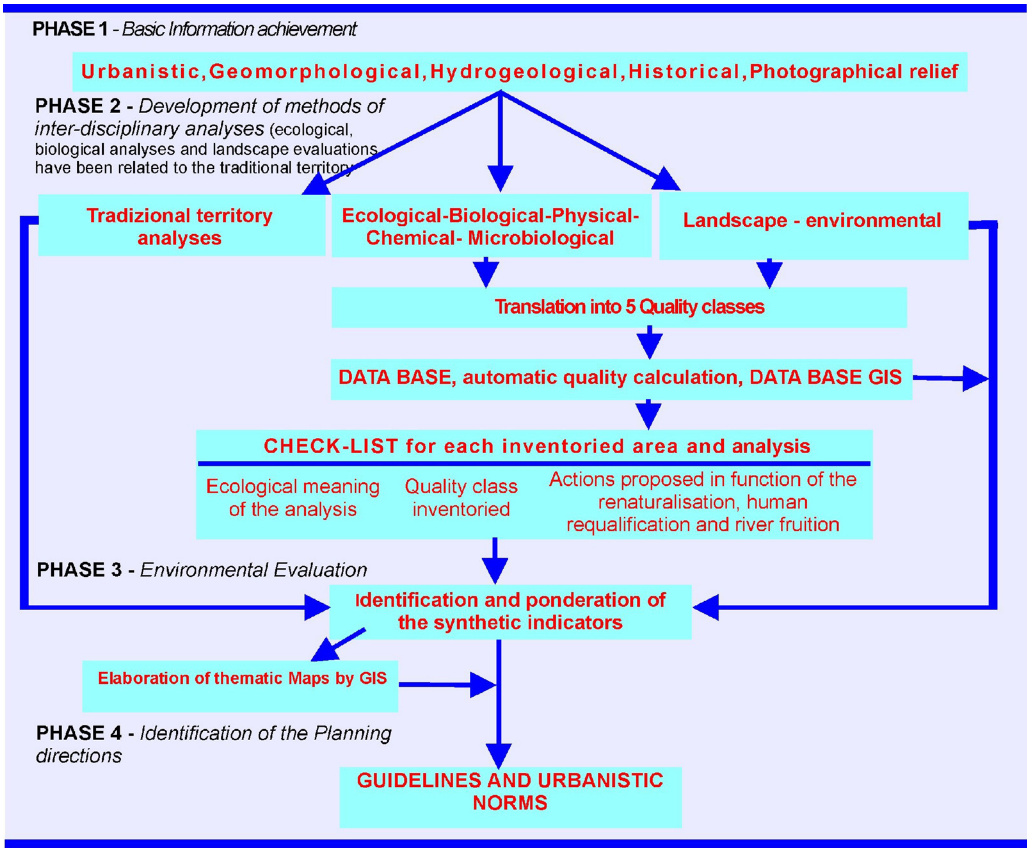

**FIGURE 1.** Scheme of the analysis and environmental evaluation (modified from [10]).

European Directive 2000/60[9] established a framework for community action in the field of water policy that has the following objectives: human development compatible with restoration, environmental safeguards, and defence from flood as well as dryness. The instrument for taking such action is the River Basin Management Plan, which outlines two essential steps: the ecological data of the river system must be read in the same context of those traditionally analysed by the Urban Planning experts, and all the "hydro-morphological and biological elements" reported in Annex V of the Water Framework Directive must present standardised monitoring procedures. It is therefore important to develop methods for monitoring the quality of ecosystem compartments so far neglected (banks and riparian areas, for example) and to define procedures for monitoring the quantitative processes of the fluvial ecosystem.

To achieve these goals, several indices for evaluating the quality of banks and riparian areas were applied within the model of analysis and environmental evaluation (Fig. 1) tested on 11 sample areas of the Adige River corridor. The procedures for translating the results of the quantitative methods into functional evaluations have also been defined. The sensitivity and the specificity of the results on the components and compartments of the analysed ecosystems have been granted. At the same time, the data were implemented together with those of the other disciplines involved in the layout of the Basin Plan. The definition of the Quality, Degradation and Risks Map is an example of actuation of an integrated reading of all the data of the river and fluvial corridor examined. The Planning Use Map, which is not reported on this paper[10,11], is an example of the norms of planning and management that can be derived from an integrated reading of the territory data.

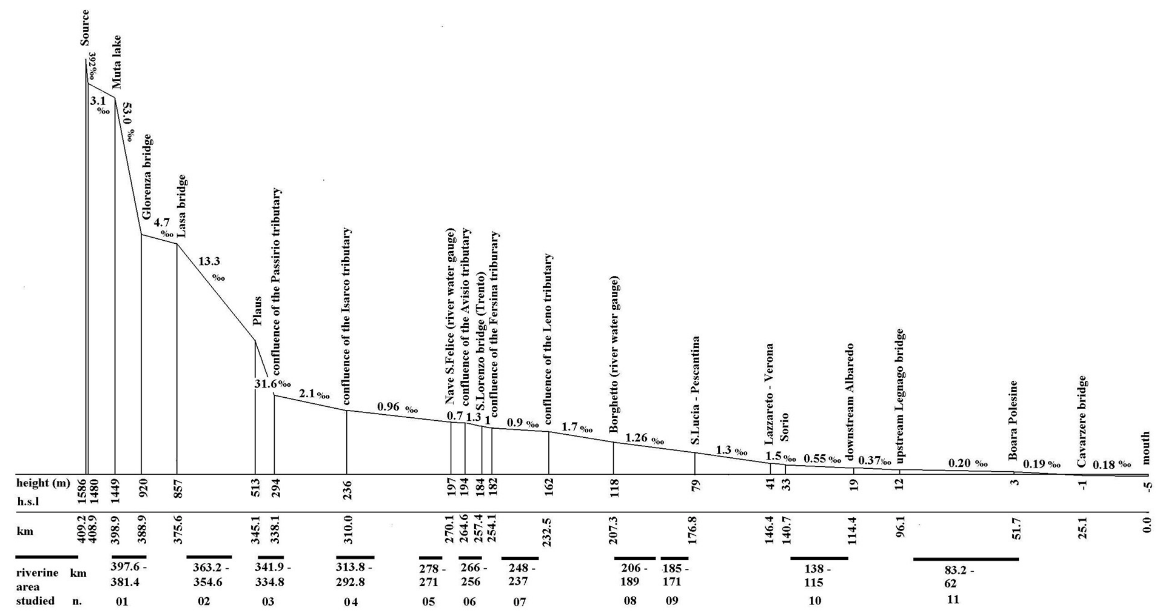

**FIGURE 2.** The Adige River. Location of the 11 areas of the fluvial corridor inventoried along the altitude-slope line (modified from [12]).

### AREA OF STUDY

According to the classification using the system A of the Annex II of the FWD, the Adige River Basin corresponds to the Ecoregion 4 (Alps) for the northern portion of its mountain basin and the Ecoregion 3 (Italy, Corse, and Malts) for its down-mountain and plain portion. The typology size based on the catchment area is "very large" (11,954 km$^2$), but "large" if we consider only the sub-basin of the Ecoregion 4, Alps (2,722 km$^2$). The Altitude typology is "high" >800 m, "middle-altitude" 200–800 m, "lowland" <200 m a.s.l., as we can show from the altitude profile of the river course (Fig. 2). The geological composition is siliceous in the higher portion of the basin and calcareous in the rest. The 11 sample areas tested in the Model of the Analysis and Environmental Evaluation also differ according to the classification using System B in the following aspects: distance from river source, energy of flow (function of flow and slope), mean water width (from few meters in the stretch 01 to more than 200 m in the stretch 11), mean water depth, mean water slope, form and shape of main riverbed (natural, embanked, channel, suspended), river discharge (flow) category, valley shape, mean substratum composition (from cobbles to sand-mud), and fallings. The landscape features differ as well as regarding the soil use of the corridor and the riparian areas and the water uses, including power, irrigation, drinking use, free time, and fish life (Fig. 3).

### METHODS

A variety of ecosystem compartments and attributes were evaluated (Table 1). Each analysis was applied within the time and space parameters required by the experimental method of the analysis itself. The translation of the results of the quantitative and functional analyses into five quality classes was achieved by a two-step evaluation that took into account the quality of the single variables or criteria considered as well as the overall quality. The intermediate evaluations put out the critical factors.

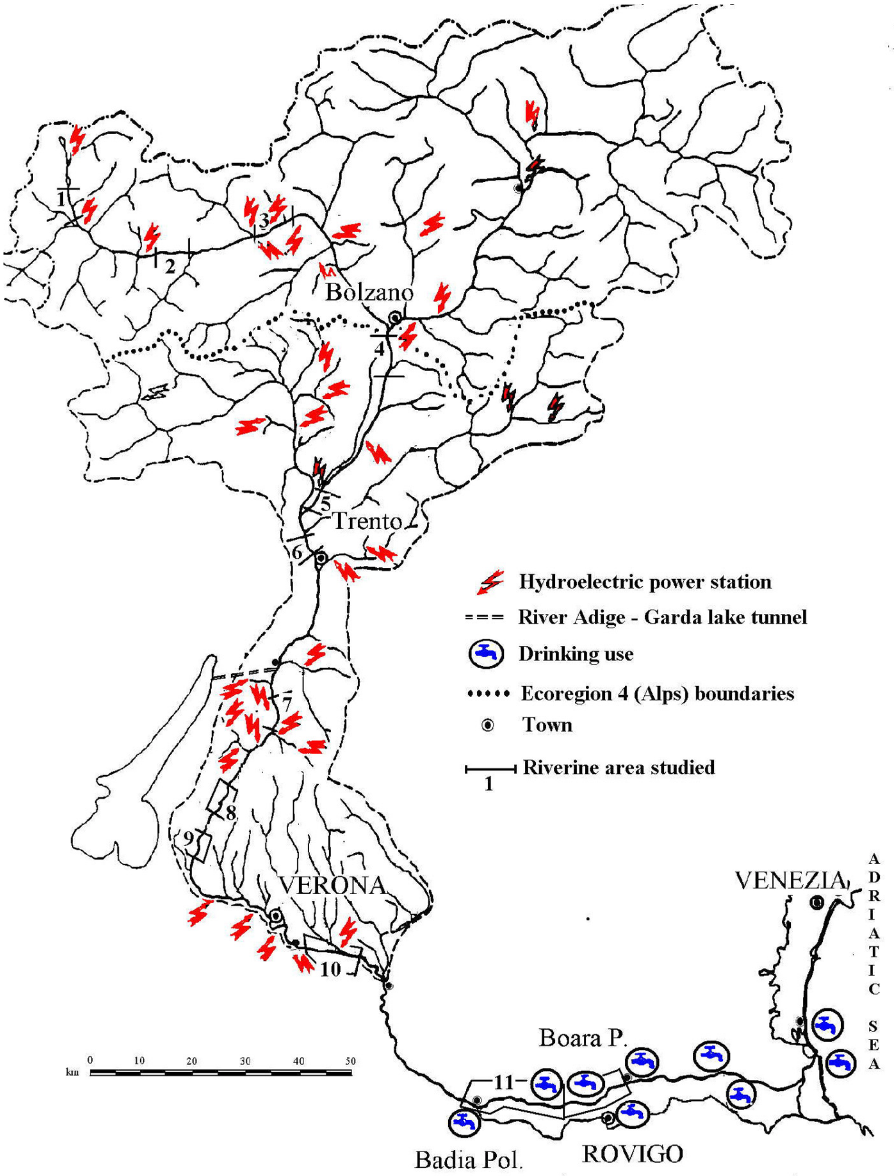

**FIGURE 3.** The Adige River Basin and the 11 areas of the fluvial corridor inventoried. Areas 8 through 11 are displayed in the square (modified from [13]).

### Assessment of the Landscape Quality of the Riparian Areas

The landscape quality of the riparian areas was evaluated by the environmental landscape index (ELI)[14]. It evaluates the *capacity for change* of the investigated areas, which can be defined as the capacity of the territory to maintain, recover, and modify its character within a perspective of the safeguard of environment and the compatibility with human activities. The ELI uses a landscape analysis form built on purpose. This form is applied within the area of fluvial pertinence (inventory area), which is defined based on the geomorphologic features and human presence within the area itself, surveyed upon existing cartography, upon aerial photos, and on-the-spot investigation. A wide inventory area can even be divided into subareas[11,13,14]. The landscape analysis programme calculates the values of the index. The database is linked to the GIS database (see Table 2; Fig. 4).

**TABLE 1**
**List of the Analyses Achieved Divided According to the Ecosystem Compartments and Components**

| River Corridor | Banks – Riparian Areas | Water Column, Bed, Interstitial Environment |
|---|---|---|
| Borderlines of single stretch of analysis, Administrative Communal, Administrative Regional | Riparian areas<br>Landscape quality: Environmental Landscape Index (ELI) | Chemical elements of surface water<br>Macrodescriptors level<br>Single uses: drinking, bathing, irrigation, Salminid water, Ciprinid water<br>Loads of nutrients |
| PRG Communal | Hydro-morphological elements:<br>Buffer strip potentiality – Buffer Strip Index (BSI)<br>Potentiality to support a high Biodiversity – Wild State Index (WSI) | Chemical Elements of groundwater:<br>Hydrochemical quality of the deep waters |
| Hydrogeological map<br>Geomorphological map<br>Geolithological map<br>Geopedological and agronomical map<br>Cultivation use map | | Biological elements<br>—Phyto and zooplankton:<br>structure and composition, abundance, biovolume, biomass, chlorophyll a, loads of animals and inorganic matter, saprobic quality, diversity indices |
| Lands agronomical classification map<br>Actual cultivation map<br>Intensive and precious cultivation map<br>Penalties in building purposes map | Biological elements:<br>Phytosociological analysis<br>Natural value of the vegetation<br>Productivity of the tree vegetation: *Salix alba, Alnus incana, Populus tremula* | —Macrozoobenthos:<br>Biological quality (EBI, Extended Biotic Index), Ephemeroptera, Plecoptera, Trichoptera (EPT) taxa, quantitative structure and composition, density, biomass, functional feeding groups, diversity indices |
| Cultural goods map<br>Communal property lands List<br>Austrian cadastre | Productivity of the grass vegetation:<br>*Phragmites australis, Typhoides arundinacea* | —Recolonisation of artificial substrata:<br>diversity, density, biomass, EPT taxa, functional feeding groups, trend of the process |
| Plan of State properties | | —Litter processing (natural leaf packs):<br>diversity and quantity of leaves, diversity and<br>quantity of macrozoobenthos |
| Table for determining the expropriation | Bank invertebrate fauna | —Interstitial fauna:<br>structure and composition, diversity<br>Hydro-morphological elements:<br>granulometry of substratum, lotic environmental quality |

Modified from [13].

### Assessment of the Banks Quality

The quality of the banks was assessed by the wild state index (WSI) and the buffer strip index (BSI)[13,14]. The BSI provides an indirect measure of the bank's capacity for filtering, metabolising, and bioaccumulating the nutrients and the pollutants conveyed by the river during the periods of high flow or percolating into the river from the surrounding territory

**TABLE 2**
**Extension of the Sampling Stretches from 8 to 11 Reported as an Example and Number of IA and Samples**

| Stretch No. | Extension km Distance from Source | Riparian Areas: ELI No. IA | Riparian Areas: Phytosociological Sampling No. | Riparian Areas: Productivity of Vegetation *Salix alba*, *Thyphoides arundinacea*, No. of Samplings | Banks: BSI | Banks: WSI |
|---|---|---|---|---|---|---|
| 8 | 209.9–219.6 | 25 | 16 | 2 sites × 6 months | 41 | 41 |
| 9 | 224.1–237.5 | 26 | 0 | | 16 | 16 |
| 10 | 271.2–294.0 | 36 | 10 | 2 sites × 6 months | 47 | 47 |
| 11 | 326.2–346.7 | 30 | 30 | 2 sites × 6 months | 29 | 29 |

The WSI reflects the potentiality of the area to support a high level of biodiversity. Both indices survey an inventory area (IA) of the bank that measures 100 × 100 m by a unique inventory sheet. The programme Banks 5.00 calculates the value and quality class of BSI and WSI. The IAs were located along the course of the river using the proportional stratified sampling method. Their number has also been increased in the fluvial stretches where the ecological analyses of the bed and the water column were carried out. Of the IA, 40% are included in the wider IA of the ELI (Table 2; Fig. 4).

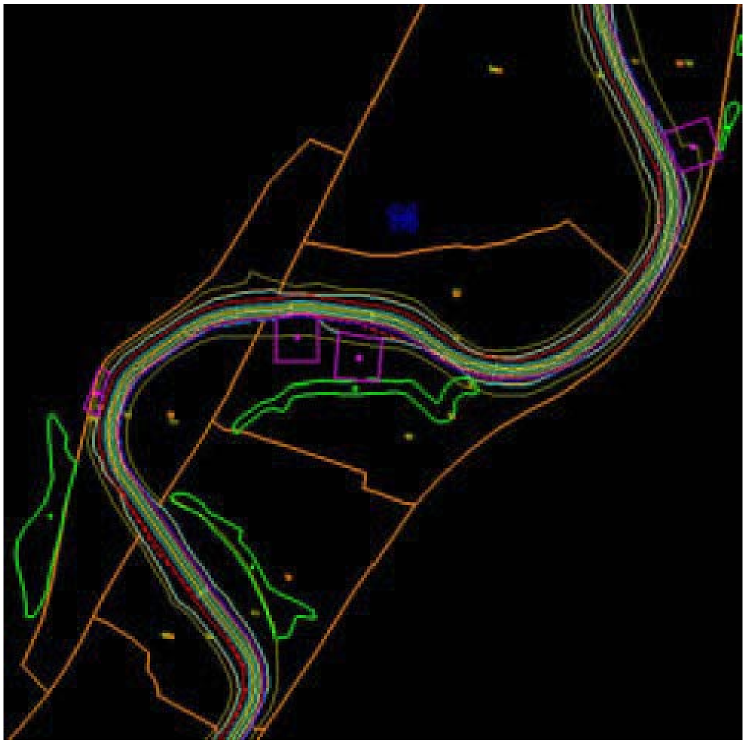

**FIGURE 4:** The GIS example; the areas of the river corridor analysed by the ELI are brown; the areas of botanical interest are green; the IA of the banks analysed by the BSI and WSI are yellow magenta. The river classes for the monitoring of the water quality according to Italian law: ecological state, macrodescriptors level, biological quality, drinkable use, bathing, Salmonid and Cyprinid suitability, irrigation[10, modified; 15].

### Quality of the Vegetation of the Riparian Areas and Fluvial Corridor

Phytosociological samplings of the riparian vegetation (Table 2) and the following 68 samplings of fluvial corridor vegetation were located according to the interpretation of aerial photos for: (1) the natural state and value of the vegetation, (2) the fluvial corridor planning, and (3) the extrapolation of productivity measures. Of the phytosociological samples, 80% were carried out in the same IA assessed for the banks quality.

The translation into five quality classes was achieved according to the following parameters: structure, vegetation typology matching the soil characteristics, percentage of presence of native species.

### Quality of the Productivity of the Riparian Vegetation

The primary production of *Salix alba* L. (riparian woods in middle and lower course) was taken into account within stretches 8, 10, and 11. *Alnus incana* (L.) Moench and *Populus tremula* L. (mixed riparian wood in higher course) were considered in stretch 2. The aboveground biomass of trees was evaluated with two complementary approaches: the biomass and the average increment of the trunk were measured by means of standard Schneider dendro-auxometric method applied to all individual trees in a 400-$m^2$ plot in each site; the biomass of branches was calculated according to an allometric model[16] as well as their increment[17]. Several linear regressions between the weight of branches (dependent variable) and diameter of the trunk (independent variable) were calculated on sample individual trees of *S. alba* representative of different diametric classes. The total biomass is the sum of biomasses of the trunk and branches.

The primary production of herbaceous riparian communities was measured in four sampling stretches within the areas near those already examined for their tree production. *Phragmites australis* (Cav.) Trin. community was taken into account in the higher course (stretch 2), and *Typhoides arundinacea* (L.) Moench communities in the medium and lower courses (stretches 8, 10, and 11). Samplings were carried out monthly. Each sampling phase consisted of cutting the aboveground plant biomass in ten 0.1225-$m^2$ randomly selected plots. Green biomass was kept in an incubator at 105°C up to constant weight. Eventually dry weight was measured and primary production calculated according to Groenondijk[18].

To translate the quantitative results into five quality classes the following criteria were adopted:

1. The results of production studies, with particular reference to the age structure of woody communities;
2. The morphological characteristics of the river course (regular or suspended riverbed);
3. The duration of contact between herbaceous vegetation and water from river and/or water table; and
4. The extension of the communities represented in the vegetation map.

### Quality of Plankton, Water Column, Sediments in the Lowland Course of the Adige River

Twelve series of samplings of phytoplankton and zooplankton were collected on three stations (11a–c): the first two corresponded to the initial and final sample stretch 11 (km 331.116–346.750). In 11c (km 357.800) the measures of the flow is recorded in continuum (Table 3).

Because the number of the sampling stations was too small, a rigid range could not be assigned to variables in the assessment of quality classes. The translation of quantitative results into five quality classes, more suitable for biomonitoring, was carried out by a double-entry table.

**TABLE 3**
**Sampling of the Biological, Physical - Chemical Elements in the Lowland Course of the Adige River**

| No. Stretch | Element Plankton | Samplings | | | | |
|---|---|---|---|---|---|---|
| | | No. in Space | Frequency | Volume | Depth (m) | Methods |
| 11a–c | Phytoplankton | 1 × every single transect | A (1 h) B × 12 months | 5 l | 0.10–0.30 | Bottle graduate |
| 11a–c | Zooplankton | 3 × every single transept | × 12 months | 50 l total | 0.10–0.50 | Patalas modified |
| 11a–c | Water | 3 × every single transect | 15 day range | 5 l | 0–0.20 | Bottle graduate |
| 11a–c | Sediment | 5 × every station | 1 × season | 20 × 20 cm | 0–0.10 | Grab |

| Biological Elements | Laboratory Analysis | References |
|---|---|---|
| Phytoplankton | Subsample, fixative, count, identification, biovolume, chlorophyll *a* | 64,65,66,67,68,69, 70,71,72,73,74 |
| Zooplankton | Copepoda, Cladocera gathered by 45-μ nets, Rotifera 25 μ, biomass (dry weight) of Rotifera, Diversity Indices, Saprobic Index, Cluster Analysis, Load organic and inorganic quantities | 15,21,22,23,30,75, 76,77,78,79 |
| Chemical physical elements | Chemical – physical, microbiological parameters of water; N and P loads in water and sediments | 80,81 |
| Hydrological elements | Continuous flow measurement | 15 |

The qualitative score of excellent, medium, or poor for every variable evaluated—variety, % plankton species, plankton species dominance, density, biovolume, Diversity index (H'), Maximum Diversity (H max), Eveness index (J), Dominance index (D), Saprobic index value, Rotifera loads, inorganic and organic loads, chlorophyll *a*, conductivity, pH, cloudiness, sedimentary solids, total dry weight, total suspended solids, stationary residuum, volatile solids—was indicated according to correspondence to the literature data related to the season and the hydrological variability[15,19,20,21,22,23,24,25,26,27,28,29,30,31,32,33,34,35].

The following criteria were taken into account when the self-purifying capacity of the lowland course of the Adige River was classified according to quality class: the increase, decrease, and constancy of the loads of N and P flowing through the three stations; and the quantity of nutrients released by the sediments.

### Quality of Interstitial Hyporheic Environment of the Rhythral Watercourse

Inquiries on the hydrochemical characteristics of the interstitial and surface waters, on the interstitial hyporheical fauna, and on the sediment granulometry were carried out within stretches 8 to 10 (Table 4).

The interstitial hyporheical fauna was sampled seasonally within stretches 2 to 10 (five pseudoduplicate for each area), by the Bou-Rouch method at a depth of 30–40 cm and filtering by a 25-μm net the 30 l of sample water.

The translation of quantitative results into five quality classes was carried out by a double-entry table. The qualitative score of excellent, medium, or poor for the single variables evaluated — variety, percentage of interstitial species, interstitial density/total density, number of

**TABLE 4**
**List of the Elements Evaluated in the Interstitial Environment in the Stretches 8, 9, 10**

| Element | Sample Dimensions | No. of Sampling | | References |
|---|---|---|---|---|
| | | In Space | In Time | |
| Hydrochemical elements of surface water | Point | 10 | 4 | 80,81 |
| Hydrochemical elements of interstitial water | Point (30- to 40-cm depth) peristaltic pump | 10 | 4 | 46,80,81 |
| Interstitial sediment granulometry | (40- × 40- × 40-cm depth) | 2 | 2 | 15 |
| Interstitial hyporheic fauna | Point (30- to 40-cm depth) | 15 | 4 | 82,83 |

Ephemeroptera, Plecoptera, Trichoptera (EPT) taxa, ratio of EPT taxa to total taxa, ratio of EPT density to total density, H', H max, J, D, Saprobic index — was indicated in conformity with the literature data and the results of the historical series of 1972–73 on the course of the Adige River[36,37,38,39,40,41,42,43,44,45,46,47,48,49,50,51,52,53,54,55,56,57,58,59].

Interstitial hyporheic water samples and surface samples were gathered at each interstitial hyporheical fauna sampling. The following parameters were analysed: temperature, conductivity, total suspended solids, total hardness, total alkalinity, nutrients (ammonia, nitrate, nitrite, total nitrogen, ortophosphate, total phosphate), biochemical oxygen demand, chemical oxygen demand, oxygen dissolved, total organic carbon, and some anions and cations (calcium, magnesium, sodium, potassium, chloride, sulfate). Moreover, the abundance of total coliform, fecal coliform, fecal streptococci and *Salmonella* was tested.

The evaluation of the quality of the interstitial hyporheic waters was carried out according to Italian Decreto Legislativo 258/2000 concerning underground waters. The evaluation of the quality of the surface waters was made according to the DL 258/2000[60] of the evaluation of the macrodescriptors pollution level.

The granulometry data on a low flow period after a medium flow period and after a long period of low flow were translated into quality classes by means of a reference range based on both the class I as proposed by Angelier[61] for gravel and granules up to 2 mm (45 to 75%) and the V class for a rhythral stretch with fine compact sand without interstices.

### Morphohydrological Quality of the Riverbed

The granulometry of the riverbed substratum was inventoried seasonally. It was translated into quality classes according to the correspondence of the mineral grain size dominance to the river type expected by the River Continuum Concept. The evaluation of the morphohydrologic characteristics of the lotic environment was carried out according to the scheme reported in Table 5, by adopting the following criteria:

1. The sampling of the substratum must be carried out during a period of lower flow (both regulated and natural).
2. The homogeneous stretch of bed of the sampling must be at least 40 m long.
3. The velocity of the flow must be measured along an entire section of the bed and possibly within the different morphological types: pool, run, and riffle.
4. "Shadowed" indicates the percentage of wet bed that is in contact with the banks, and that falls in the shadow cone induced by the riparian vegetation.
5. The reference substrata in the rhythral stretches include stones, cobbles, rough gravel, medium gravel, fine gravel, sand, mud, rough and fine organic particles.

**TABLE 5**
**The Lotic Environmental Quality Class as Determined by the Mean of the Single Classes**

| Class | Hydrological Variations | No. of Substrata–Extension | Diversity of Velocity Flow | % Shadow |
|---|---|---|---|---|
| I | Natural | 7 Types–similar | Excellent | > 50 |
| II | Regulated, nonrelevant | 5 Types–similar | Good | = 50 > 30 |
| III | Regulated, modify the low flood | 3 Types–different | Medium | = 30 > 10 |
| IV | Regulated, weekly | 2 Types–dominate 1 | Scarce | = 10 |
| V | Regulated, daily | 1 Type | Laminar | Absent |

The reference substrata in the potamal stretches includes floating macrophytes, rooted macrophytes, rough sand, fine sand, mud, rough and fine organic particles.

### Quality of Macrozoobenthos

The inquiries on macrozoobenthos were particularly articulated and took into consideration the structure of the community (EBI)[62,63], the quantitative aspects of the taxonomic and functional feeding groups, the main function of the leaf litter breakdown (natural and artificial), and the capacity for colonising inorganic substrata.

Table 6 reports the methods and the time and space dimension of the qualitative and quantitative analyses carried out on the macrobenthic communities of the Adige River in stretches 8 to 11. See Table 7 for a translation of the qualitative sample data on the macrobenthic fauna into a functional evaluation, which is more useful for the survey and control monitoring.

**TABLE 6**
**Inquiries on Macrobenthos Referring to the Method Used, Number of Duplicate Samples, and Laboratory Analyses**

| Inquiry | Tools | Ref. |
|---|---|---|
| Qualitative structure and composition of benthos | Net | 63 |
| Density and biomass of benthos | Surber | 84 |
| Leaf litter breakdown processing | Natural leaf packs | 85,86 |
| Leaf litter breakdown processing | Artificial leaf – bags of *Alnus glutinosa* | 87,88,89 |
| Colonisation | Boxes | 90,91 |

| Analysis | Environment | Dimensions | No. of Duplicates In space | No. of Duplicates In time |
|---|---|---|---|---|
| Qualitative benthos | Substratum | 20–50 m | 1 | 4 |
| Density benthos | Substratum | 0.2 × 0.22 m | 2–3 | 4 |
| Litter – processing | Natural leaf packs | 0.1 × 0.1 m | 5 | 3 |
| Litter – processing | A. glutinosa *leaf – bags* | 0.15 × 0.15 m | 5 | 6 |
| Colonisation | Artificial substrata | Area 26.5 × 36.5 m; depth 20 cm | 5 | 6 |

| Laboratory Analysis | References |
|---|---|
| Identification taxa, density for $m^2$ | 92,93,94 |
| Functional feeding groups, density for $m^2$ | 95 |
| Dry weight biomass for $m^2$ | HETO Drywinner G-55 at the Biol. Lab. Laives (BZ)[96] |
| H’, H max; J, D indices calculation | 26,76,77 |

**TABLE 7**
**Translation of the Qualitative Results on the Macrobenthic Fauna into Quality Classes**

| Quality Class | Variety (No. of Taxa) | EPT Taxa/Total Taxa | EBI | Score |
|---|---|---|---|---|
| I | 16 | 44 | >10 | Excellent |
| II | 11–15 | 44–36 | 8–9 | Good |
| III | 6–10 | 36–30 | 6–7 | Medium |
| IV | 2–5 | 30–20 | 4–5 | Scarce |
| V | 0–1 | <20 | <4 | Very bad |

**TABLE 8**
**Method for Translating the Quantity Data for Macrobenthos into Functional Evaluations: Step 1**

| Quality Class | Variety (No. of Taxa) | Density (n/m$^2$) | % Density EPT taxa (n/m$^2$) | Biomass (mg/m$^2$) | Contribution in Biomass of Functional Feeding Groups |
|---|---|---|---|---|---|
| I | >12 | > 10.000 | >51 | >2.000 | dg <60% and 5 gp |
| II | 10 to 11 | 5.000–9.999 | 41–50 | 1.000–1.999 | dg 61–70% and 5 gp |
| III | 8 to 9 | 1.000–4.999 | 21–40 | 500–999 | dg 71–80% and 5 gp |
| IV | 5 to 7 | 500–999 | 10–20 | 100–499 | dg 80–89% and/or 4 gp |
| V | <5 | < 499 | <10 | <100 | dg >90% and/or 3 gp |

| Quality Class | Diversity (H’) | Homogeneity (H max) | J (H’/Hmax) | Richness (D) |
|---|---|---|---|---|
| I | 4 ≥ H'≤ 5 | 4 ≥ H max ≤ 5 | 0.8 ≥ J ≤ 1 | 4 ≥ D ≤ 5 |
| II | 3 ≥ H' < 4 | 3 ≥ H max < 4 | | 3 ≥ H' < 4 |
| III | 2 ≥ H' < 3 | 2 ≥ H max < 3 | 0.6 ≥ J < 0.8 | 2 ≥ H' < 3 |
| IV | 1 ≥ H' < 2 | 1 ≥ H max < 2 | | 1 ≥ H' < 2 |
| V | H' < 1 | H max < 1 | J < 0.6 | H' < 1 |

dg = percentage of the biomass of dominant groups compared to the total biomass.
gp= number of the functional feeding groups occurring in the sample.

**TABLE 9**
**Method for Translating the Macrobenthos Quantity Data in Functional Evaluations: Step 2**

| Quality Class | Score × Analysis | Total Score | Quality |
|---|---|---|---|
| I | 5 | 37–45 | Excellent |
| II | 4 | 28–36 | Good |
| III | 3 | 19–27 | Medium |
| IV | 2 | 10–18 | Scarce |
| V | 1 | 9 | Very bad |

The procedures for translating the data, obtained by quantitative samplings of macrobenthos and by the analyses of the organic (natural leaf packs, *Alnus glutinosa* leaf bags) and inorganic substrata colonisation into five quality classes are the same in the first step (Table 8). When in the second step the overall quality class was defined, results from the following analyses were assessed: natural leaf packs, artificial leaf bags, inorganic substrata, and the trend of the process (Table 9, 10).

**TABLE 10**
**Method for Translating the Macrobenthos Quantity Data of the Natural Leaf Packs, Artificial Leaf Bags, and Inorganic Substrata in Functional Evaluations: Step 2**

| Quality Class | Total Score | Quality | Trend of the Process |
|---|---|---|---|
| I | 37–45 | Excellent | A = under expectation |
| II | 28–36 | Good | B = unexpected, for natural progression |
| III | 19–27 | Medium | C = very unusual for time progression |
| IV | 10–18 | Scarce | |
| V | 9 | Very bad | |

### Implementation of Results

According to the analysis and environmental evaluation model defined and applied on the 11 stretches of the Adige River and the fluvial corridor, the biological and ecological results were implemented and inserted in the GIS of the Adige Basin Authority, by the following four themes:

1. Classification of the surface water quality as requested by the Italian norms for each use;
2. Definition of the ecological status as requested by the DL 258/2000;
3. Evaluation of the ecological status according to the Elements of the Annex V of the FWD 60/2000, using also the results of functional evaluations;
4. Elaboration of the Quality, Degradation and Risk Map.

## RESULTS AND DISCUSSION

### Classification of the Surface Water Quality as Requested by Italian Norms

In sample stretches 8 to 11, swimming, salmon breeding, and irrigation are completely forbidden (see Table 11). These areas can be used for drinking only after treatment through high costs of plant and management. Stretches 8 to 10 can host Ciprinida only; stretch 11 is unsuitable.

### Definition of the Ecological Status

As shown in Table 12, the ecological status of the Adige River (as defined by the method proposed by DL 258/2000) corresponds to the lowest level among the classes of the macrodescriptor level of pollution (MLP) and of biological quality (BQ) as defined by EBI. In addition, it shows a general degradation.

**TABLE 11**
**Classification of Superficial Waters According to Use**

| | | Water Life | | Irrigation | | |
|---|---|---|---|---|---|---|
| Stretch | Drinking | Salmonida | Ciprinida | Microbiology | Chemistry | Swimming |
| 8 | A3 | Unsuitable | Suitable | Unsuitable (contaminated by *Salmonella)* | I | No |
| 9 | A3 | Unsuitable | Suitable | Unsuitable (contaminated by *Salmonella)* | I/II | No |
| 10 | A3 | Unsuitable | Suitable | Unsuitable (contaminated by *Salmonella)* | I/II | No |
| 11 | A3 | Unsuitable | Unsuitable | Unsuitable on not cooked vegetables | II | No |

A3 = appropriate physical and chemical treatment, including affination and desinfection, is needed to make the water fit to drink.

**TABLE 12**
**Ecological Status Classification of Stretches 8 to 11**

| Stretch | Water Samples | MLP | Benthos Samples | BQ | Ecological Status |
|---|---|---|---|---|---|
| 8 | 8 | II | 20 | III/II | III/II |
| 9 | 8 | II | 20 | III | III |
| 10 | 8 | II | 12 | IV | IV |
| 11 | 36 | II | 12 | IV/III – V | IV/III – V |

This monitoring is useful for identifying the quality objectives that need to be achieved, but it does not offer the knowledge necessary to determine the possible structure and management interventions necessary to reach those objectives. It does not offer planners the information they need to identify effective and coherent interventions and make appropriate decisions.

### Assessing the Ecological Status

The biological, ecological, and landscape analyses previewed by the model of analysis and environmental evaluation proposed for and applied on the 11 stretches of the Adige River allowed an assessment of their ecological status. See Fig. 5 for a synthetic comparison of the landscape quality and the natural state and value of vegetation of the riparian areas, as well as the potentiality of the banks to sustain a high level of biodiversity and filter and bioaccumulate nutrients and pollutants within the stretches 8 to 11. The primary production of woody riparian vegetation is synthesized in Tables 13 and 14, and the production trend of herbaceous vegetation is shown in Table 15 and Fig. 6. Tables 16 and 17 report the synthesis of the functional evaluations on interstitial hyporheic and benthic fauna, on plankton, and on self-purifying capacity (only for stretch 11), for 30 km ca. of the lowland watercourse.

#### *Fluvial Corridor Quality of the Adige River in Sample Stretches*

The landscape quality of the riparian areas is mostly medium. There are a few good quality areas of class I. Human pressure in stretch 9 is deep, as shown by the prevailing presence of class III, IV, and V IA. The result is confirmed by the absence of areas with vegetation that is typical from a phytosociological point of view. The discordance shown in Fig. 5 between the low percentage of IA in class I and II and the high percentage of vegetation relevance in class II within stretches 8, 10, and 11 is caused by the different surface sampled in the two methods. Both confirm the presence of areas in which the natural value of the vegetation is medium or presents the typical typology (class I).

In these areas the tree production of *Salix alba* and grass production of *Typhoides arundinacea* shows a class II quality. The first result is confirmed by the diametric structure of the tree communities and wood production results (Table 13, 14). The highest values were surveyed despite the differences in the structure of the populations. The highest wood biomass occurs in stretch 8 where the oldest individuals of *S. alba* occur. The highest growth values are found in stretch 10, where the tree population is dominated by young individuals.

The wood in stretch 2, although having a young-tree dominating structure, shows low growth values due to bad soil composition (abundant gravel fraction) if compared with the other three sites in which sand-silt soil textures prevail. (These data on stretch 2 is not reported in this paper.)

The herbaceous riparian communities also show the highest values in these stretches, even if few differences occur that show the influence of the flow regimen on their primary productivity. As shown in Table 15 and in Fig. 6, two seasonal patterns of biomass occur: in stretches 8 and 10

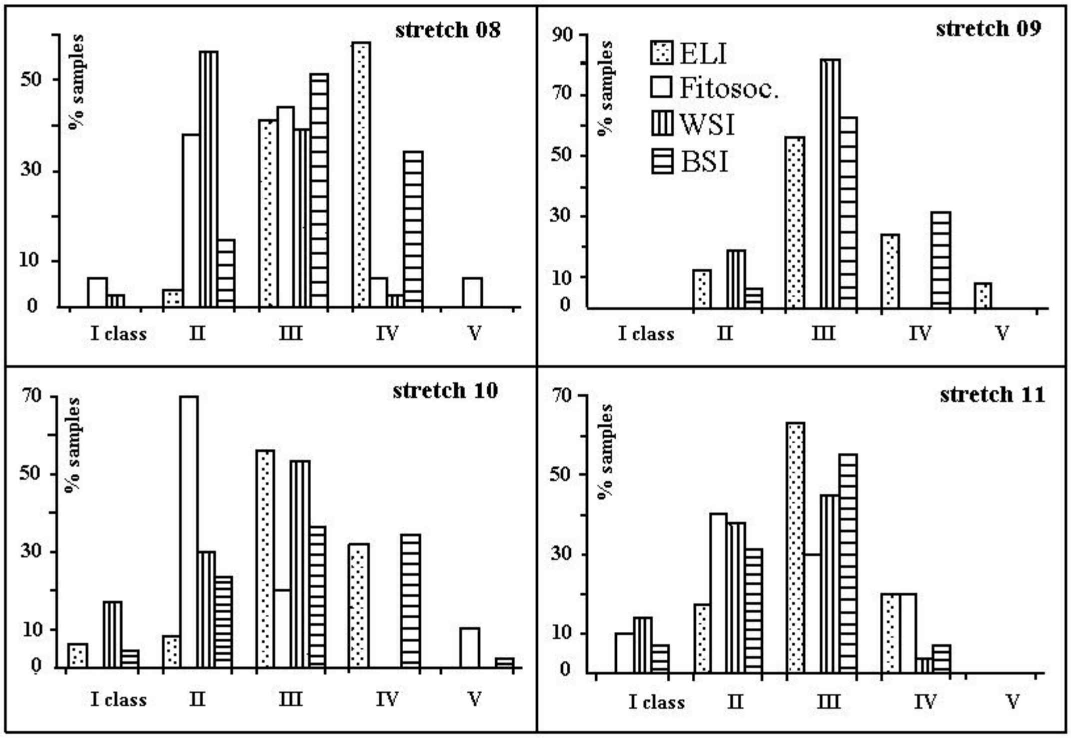

**FIGURE 5.** Trends of percentage of the riparian areas, riparian vegetation, and banks wild state – buffer quality class.

### TABLE 13
## Biomass of Trees and Their Yearly Growth in a Hectare

| Stretch | Biomass of Trunk (t/ha) | Branches Biomass (t/ha) | Total Biomass (t/ha) | Yearly Growth of Trunk (t/ha year) | Yearly Growth of Branches (t/ha year) | Total Yearly Growth (t/ha year) |
|---|---|---|---|---|---|---|
| 2 | 18.46 | 1.28 | 19.74 | 6.06 | 0.22 | 6.28 |
| 8 | 93.54 | 6.59 | 100.12 | 15.13 | 0.67 | 15.80 |
| 10 | 48.80 | 2.79 | 51.59 | 18.74 | 0.46 | 19.20 |
| 11 | 84.36 | 4.42 | 88.77 | 13.65 | 0.61 | 14.25 |

### TABLE 14
## Distribution of Individual Trees within Diametric Classes

| Stretch | Species | Diametric Classes (cm) | | | | | |
|---|---|---|---|---|---|---|---|
| | | ≤5 ≤7 | >7 ≤10 | >10 ≤15 | >15 ≤20 | >20 ≤25 | >25 |
| 11 | *Salix alba* L. | 50 | 42 | 41 | 8 | — | — |
| 10 | *S. alba* L. | 55 | 59 | 18 | 2 | — | — |
| 08 | *S. alba* L. | 24 | | 20 | 24 | 4 | 1 |
| 02 | All | 46 | 19 | 13 | — | — | — |
| | *Populus tremula* L. | 16 | 13 | 8 | — | — | — |
| | *Robinia pseudacacia* L. | 8 | 2 | 4 | — | — | — |
| | *Alnus incana* (L.) Moench | 13 | 3 | — | — | — | — |
| | *Betula pendula* Roth | 5 | 1 | — | — | — | — |
| | *Prunus avium* L. | 3 | — | 1 | — | — | — |
| | *Salix alba* L. | 1 | — | — | — | — | — |

**TABLE 15**
**Net Production of Herbaceous Vegetation**

| Stretch | Yearly Total Net Production (g•m$^{-2}$) |
|---|---|
| 2 | 932.33 |
| 8 | 979.66 |
| 10 | 1168.73 |
| 11 | 1263.35 |

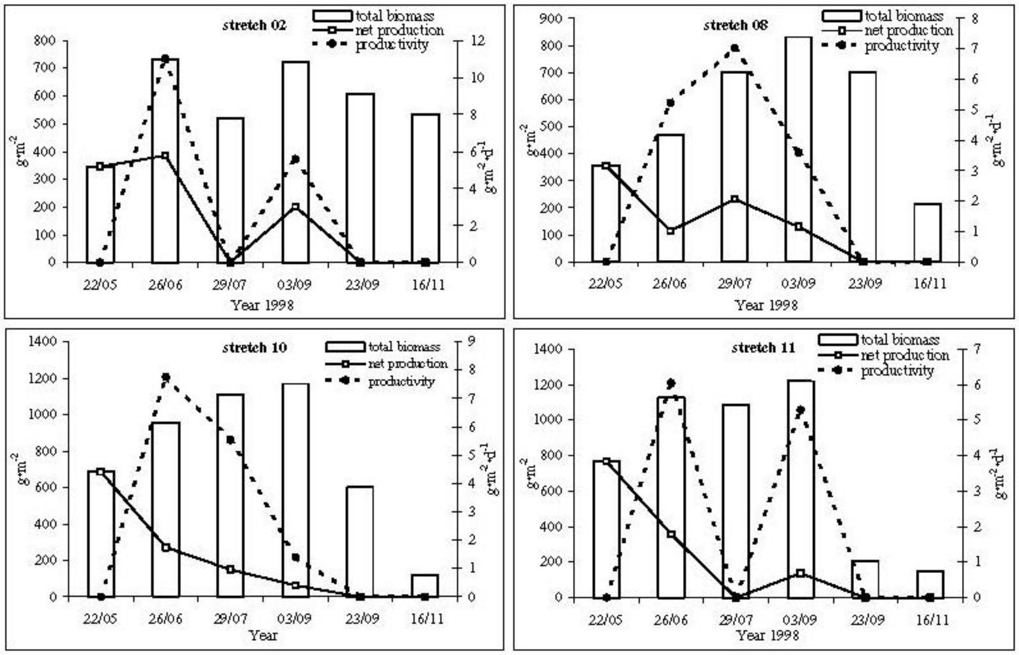

**FIGURE 6.** Primary production trends for herbaceous vegetation in the four sampling sites.

the total biomass reaches the highest value in summer; in stretches 2 and 11 two maximum total biomass values are present in early and late summer while a minimum is shown in the third sampling cycle. In the first two sites the herbaceous vegetation is submerged during floods but is protected from the destructive impact of river flows. On the contrary, in the other two sites the herbaceous vegetation is submerged during floods, but is also affected by partial destruction of aboveground parts. Both dominating species react successfully to this crisis by being deeply rooted in the soil, although their stems are prostrate after floods: *Phragmites australis* resumes its vertical stem growth, while *T. arundinacea* produces new shoots from the stem nodes. Production values of *T. arundinacea* communities are generally greater in the lower course of the Adige River if compared to alpine course. *P. australis* shows higher values in the highest site, but its production values are quite small if compared to those measured in lacustrine reedbeds or on the banks of slow-flow watercourses where little or no disturbance occurs.

The banks show a potentiality to support a high biodiversity, good-medium (classes II to III) (Fig. 5) where a strip of vegetation, even very small, stays between the river and the agrarian cultures. The wild state quality increases in the banks of the riparian areas where ELI and the natural value of the vegetation show class I quality. The capacity to filter and bioaccumulate nutrients and pollutants conveyed downstream during floods and/or coming from the territory is almost absent; in some stretches the banks could favour increased pollutant concentrations. The IA of class I represent very small banks stretches.

**TABLE 16**
**Loads of Rotifera and Organic Matter in the Water Column, and Quality**

| | km 331 | km 346 | km 347 |
|---|---|---|---|
| ***Rotifera dry weight kg/day/year*** | 21 | 23 | 26 |
| Organic matter dry weight t/day/year | 47 | 51 | 50 |
| Plankton | | III/IV class of quality | |
| Self-purifying capacity | | IV class of quality | |

**TABLE 17**
**Quality Class of Components, Compartments, and Functional Processes**

| Sample Stretch | Inter-stitial Water | Inter-stitial Fauna | Interstitial Substratum Granulometry | Lotic Environ-ment | Macro-benthos (Quantity) | Natural Leaves Breakdown | Leaf Bags Break-down | Leaf Bag Colonisa-tion | Colonisation of Artificial Substrata |
|---|---|---|---|---|---|---|---|---|---|
| 08 | II | III | III | III | III to II | | A to A | C to IV | A IV to III |
| 09 | II | III | III | II | III to IV | III to III | A to A | D to IV | A III |
| 09 | II | III | | IV | III to IV | | | | |
| 09 | II | III | | III | III to IV | | | | |
| 10 | | IV | | IV | | | | | |
| 10 | | | | IV | III to IV | | | | |
| 11 | | | | IV/III | III to IV | | E to D | B to II | |
| 11 | | | | IV | III to IV | | | | |

### *Water Column in the Plain Stretch of the Adige River*

The absence or/and reduction of the banks' buffer capacity are confirmed by specific research on the nutrient loads conveyed into the water column and occurring in the sediments along the 30 km of the plain stretch corresponding in the first 20 km to the stretch 11. The evaluation of the self-purifying capacity of the river achieved according to these data shows a class IV. The regulation of the banks and the flow are major factors in preventing the development of a plankton community (phytoplankton and zooplankton) that can contribute to the water column's self-purification (class III/IV). Other results confirm this fact: phytoplankton presents only one peak in the winter months during the continual low flow regimen even if nutrients never limit its development. Chlorophyll and pheophytin, and zooplankton and Rotifera show a similar trend. Rotifera is the dominant group as in the zooplankton of the Po River, but the percentage of planktonic species and the loads of Rotifera as dry weight are very low (see Table 16).

### *Riverbed, Interstitial Environment, and Macrobenthos*

The synthesis of the functional evaluations on the interstitial hyporheic and macrobenthic fauna shown in Table 17 is useful for understanding the level of the degradation already shown by the EBI. Not only are the structure and the composition of macrobenthos compromised, but also the biological processes. The morphological conditions of the riverbed, often bearing periodic and drastic excavations of bed and banks, and the flow trend—which is very regulated, with weekly and even daily variation caused by power management—have modified the macrobenthos as well as the interstitial hyporheic fauna and the granulometric structure of the superficial and deep bed: the riverbed became bold and waterproof, with a significant loss of microhabitats for colonisation.

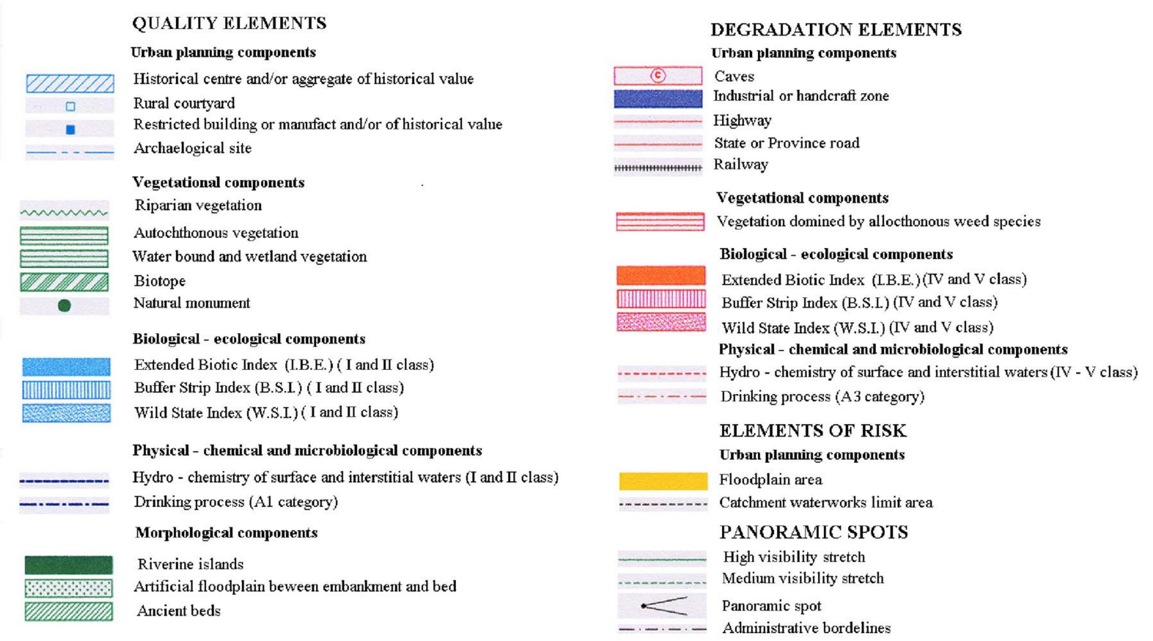

**FIGURE 7.** Map of quality, degradation, and risks (modified from [10]).

### Quality, Degradation, and Risks Map

All the results of the qualitative and quantitative monitoring are introduced in the database of GIS. These data together with the human impact elements of the fluvial corridors shown in Table 1 are elaborated according to a five-dimensional view into the planning and management of the Adige River Basin. The thematic map showing quality, degradation, and risks presented in Figs. 7, 8, and 9 is a example of the higher precision achieved when the following are taken into account: the more affected components of the ecosystem or those to be safeguarded; the potential fruition of the different stretches of the river supported by the integration of the biological-ecological data with those deriving from the planning process[11].

### CONCLUSIONS

Monitoring waters to classify them for each single use and to define their ecological status is useful for identifying water quality objectives to be achieved. However, monitoring methods do not offer enough data to show the possible structural and management interventions necessary to reach the fixed quality objectives. The procedures that this paper propose, which use multiarticulated ecological indices and functional evaluations, provide a synthetic, representative view of the structural and functional aspects of the river ecosystem that planners can easily interpret. These innovative procedures evaluate the biological, ecological, and hydromorphologic data of the river, the banks, and the riparian areas, along with the human impact elements of the fluvial corridors. They provide much more precise information on the interventions and actions needed to improve the quality of the watercourses in its complex and to obtain fixed quality objectives.

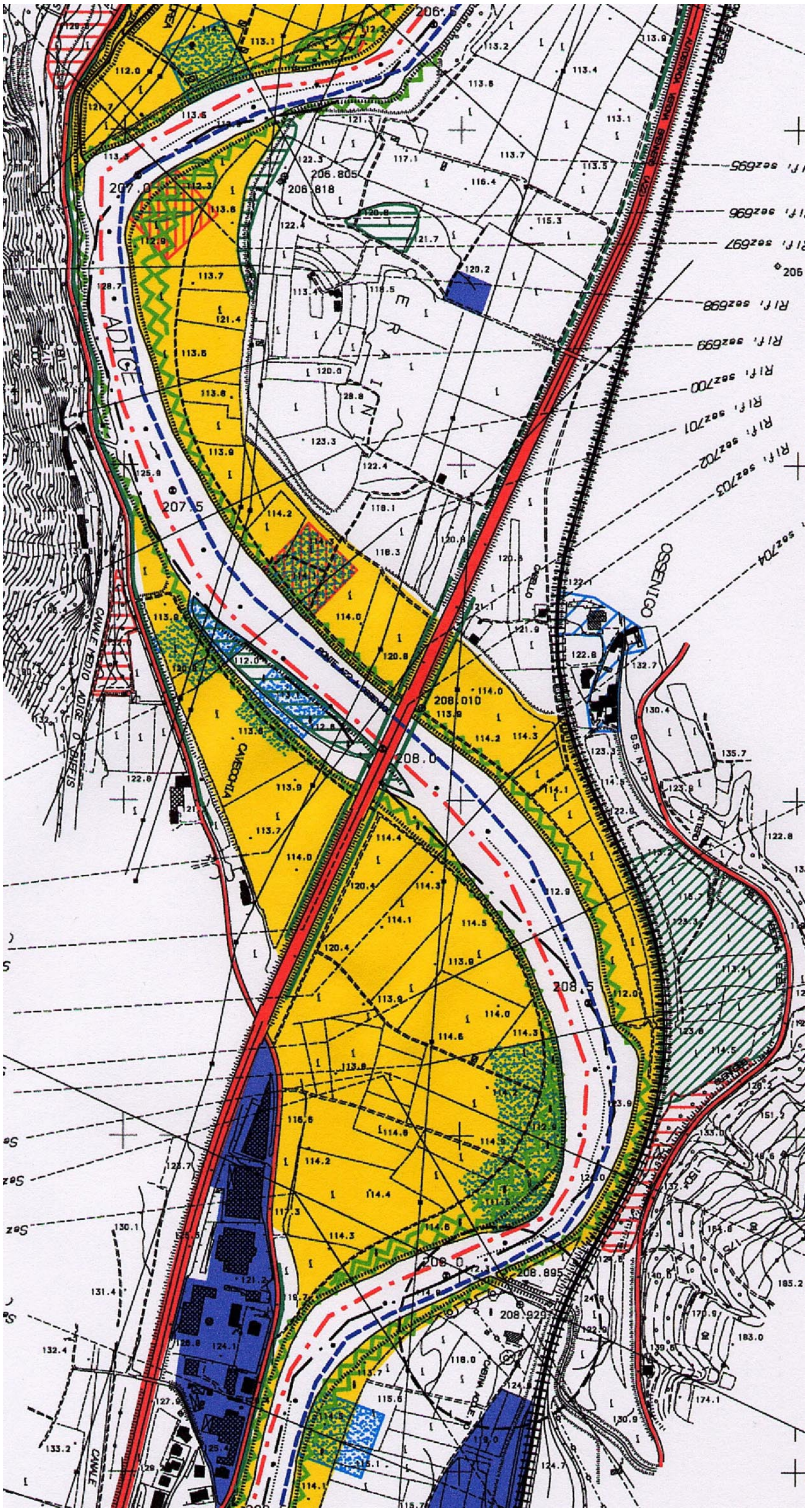

**FIGURE 8.** Map of quality, degradation, and risks for stretch 8 (modified from [10]).

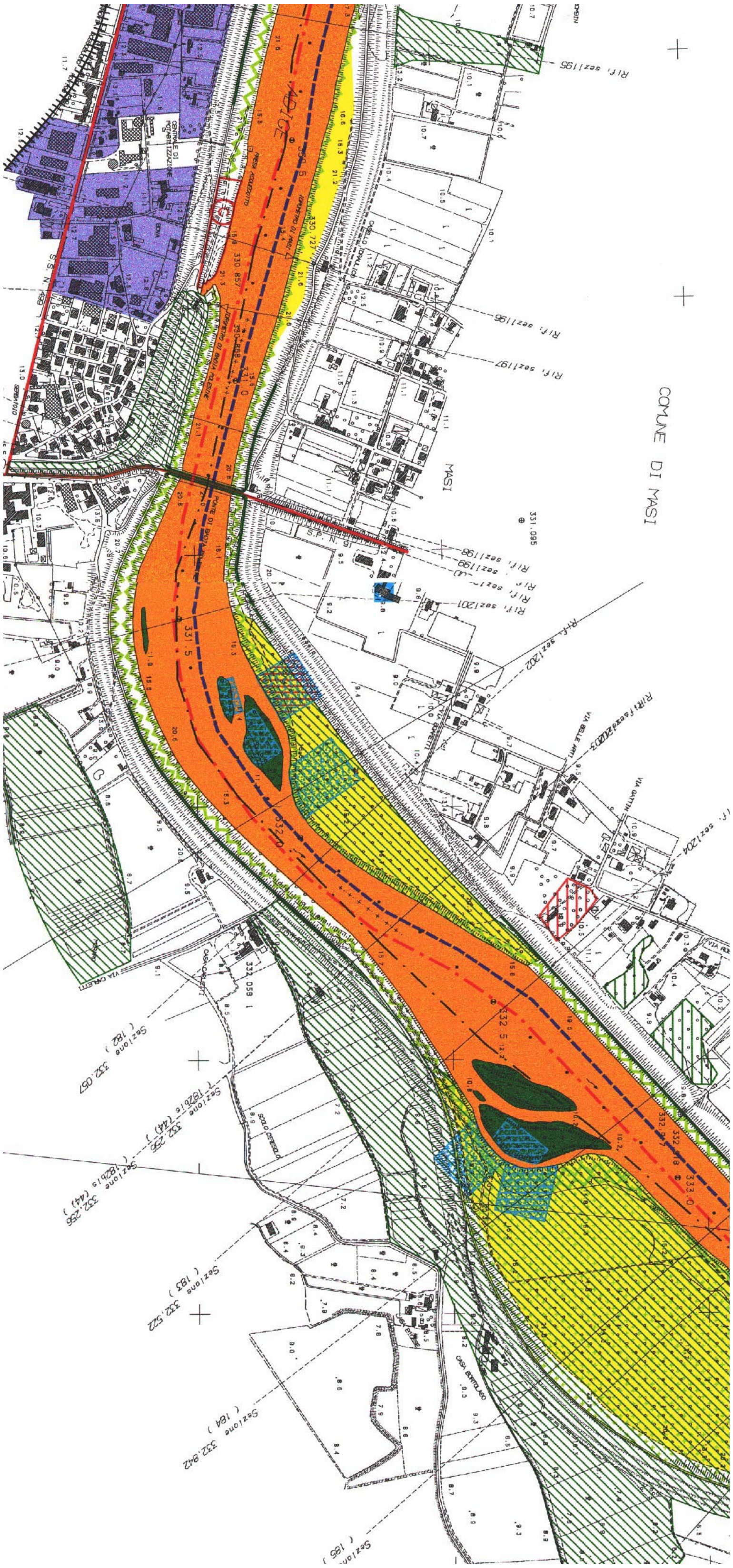

**FIGURE 9.** Map of quality, degradation, and risks for stretch 11.

### ACKNOWLEDGEMENTS

This paper relies on research and studies that were financed by the National Basin Authority of the Adige River to support the layout of the Adige Basin Management Plan.

### BIOSKETCHES

**Maria Giovanna Braioni** is Associate Professor of Zoology, Teacher of Conservation of the nature and its resources and of Ecology of Lotic Environments at the Department of Biology of the University of Padua. Her research interests include evaluation of the structure and function of macrobenthos, plankton, interstitial fauna, and of the quality and function of the riparian environment. Since 1990 she has been in charge of multi-disciplinary researches aimed at the rivers restoration.

**Anna Braioni** is an architect, associated to the National Institute of Urban Planning (INU). She was co-director of Major’s thesis in Architecture and Applied Ecology. Her research interests include eco-compatible planning, projects of evaluation, rehabilitation and restoration of the Environment and the Landscape.

**Loredana Girelli** is an architect, with ressearch interests in the evaluation of the fluvial landscape.

**Francesco Bracco** is Associate Professor of Environmental and Applied Botany at the Faculty of Pharmacy of University of Pavia. He holds a Ph.D. in Systematics and Vegetal Ecology (Geobotany). Prof. Bracco’ research interests include phytosociological study of the vegetation of Po Plain (N-Italy) with special reference to freshwater and fluvial habitats and planning and management of phytosociological databases for the vegetation of Italy (project LISY of Italian Association for Phytosociology).

**Mariacristina Villani** is a Post-doctoral fellow at Biology Department of Padova University. She holds a Ph.D. in Geobotany. Her research interests include phytosociological studies of fluvial habitats of NE Italy, production studies of riparian plant communities, floristic and vegetation analyses of Euganean Hills (NE of Italy), and monitoring control of forests.

**Gianpaolo Salmoiraghi** has 26 years experience in river ecology. He has been senior scientist since1992 and Lecturer in Hydrobiology and Applied Ecology from 1992, at Biology Department of Bologna University. His major topics are use of biotic indexes in environmental monitoring, functional aspects of macrozoobenthos community, the effects caused by dams on macroinvertebrate communities, secondary production of invertebrate communities, and breakdown and colonization processes of natural and artificial leaf packs. In 1993 he was EU consultant (DG XI) for ANNEX I 92/43/CE. He is tutor and supervisor of Ph.D. students in ecology at the University of Bologna. Since 1993 he has beren in charge of hydrobiological studies for national and regional authorities.
